# Predicting Elastic Constants of Refractory Complex Concentrated Alloys Using Machine Learning Approach

**DOI:** 10.3390/ma15144997

**Published:** 2022-07-18

**Authors:** Uttam Bhandari, Hamed Ghadimi, Congyan Zhang, Shizhong Yang, Shengmin Guo

**Affiliations:** 1 Department of Mechanical and Industrial Engineering, Louisiana State University, Baton Rouge, LA 70803, USA; ubhand2@lsu.edu (U.B.); hghadi1@lsu.edu (H.G.); sguo2@lsu.edu (S.G.); 2 Department of Computer Science, Southern University and A&M College, Baton Rouge, LA 70813, USA; congyan.zhang@sus.edu

**Keywords:** complex concentrated alloys, elastic constants, random forest regressor, gradient boosting regressor, XGBoost regression

## Abstract

Refractory complex concentrated alloys (RCCAs) have drawn increasing attention recently owing to their balanced mechanical properties, including excellent creep resistance, ductility, and oxidation resistance. The mechanical and thermal properties of RCCAs are directly linked with the elastic constants. However, it is time consuming and expensive to obtain the elastic constants of RCCAs with conventional trial-and-error experiments. The elastic constants of RCCAs are predicted using a combination of density functional theory simulation data and machine learning (ML) algorithms in this study. The elastic constants of several RCCAs are predicted using the random forest regressor, gradient boosting regressor (GBR), and XGBoost regression models. Based on performance metrics R-squared, mean average error and root mean square error, the GBR model was found to be most promising in predicting the elastic constant of RCCAs among the three ML models. Additionally, GBR model accuracy was verified using the other four RHEAs dataset which was never seen by the GBR model, and reasonable agreements between ML prediction and available results were found. The present findings show that the GBR model can be used to predict the elastic constant of new RHEAs more accurately without performing any expensive computational and experimental work.

## 1. Introduction

RCCAs are refractory complex concentrated alloys with complicated compositions [1]. Unlike the more restricted refractory high-entropy alloys (RHEAs), RCCAs may include less than four main elements in the composition, have multiple phases, and have small entropy configurations. Compared to typical superalloys, RCCAs have recently attracted increasing interest as a potential choice for high-temperature structural application materials. This is because the appealing qualities of RCCAs which include outstanding high-temperature strength, ductility, high melting temperature, and oxidation resistance [2,3,4,5,6]. The mechanical and thermodynamic properties of alloys are directly linked to their elastic constants. For example, an elastic strain of materials under different stresses depends on the elastic constant [7]. The elastic constants are also linked to essential characteristics, including bulk modulus, shear modulus, Young’s modulus, Poisson’s ratio, and Debye temperatures. The Voigt–Reuss–Hill (VRH) averaging approximations can be used to estimate them [8].

Several theoretical density functional theory (DFT) studies [9,10,11,12,13,14] and experimental work [15,16,17] are performed to obtain the elastic characteristics of RHEAs. However, understanding of elastic constants of body-centered cubic (BCC) of RCCAs is still restricted because of a lack of high-efficiency data generation methods. It is very time consuming, expensive, and complicated to synthesize and measure the elastic constants of alloys experimentally. Additionally, computational methods need many CPU hours, making them relatively expensive for large supercells. In the literature, resonant ultrasound spectroscopy is also reported as a decent choice to measure the elastic constants of materials. However, this experimental technique has its own limitations [18]. Therefore, to accelerate the designing of RCCAs with desired properties, it is essential to adopt a time-saving and low-cost method that can efficiently explore the elastic properties of novel RCCAs. Machine learning (ML) is a reasonably easy and cost-effective tool for quickly identifying hidden patterns in a dataset. The recent trend of applying ML models in designing HEAs is promising in determining the phase formation [19,20,21,22,23,24,25] and predicting mechanical properties [26,27,28]. ML is also applied in the biomedical field to design metallic implants [29,30,31]. Wen et al. [32] used the ML approach to identify an AlCoCrCuFeNi HEA system with high hardness. Meanwhile, they also experimentally synthesized the alloy to validate their hardness prediction. Klimenko et al. [33] predicted the strength of AlCrNbTiVZr RHEA using a support vector ML model with experimental validation. Using a random forest model, the Kwak group [34] predicted various mechanical properties, such as tensile strength, nanoindentation hardness, and elongation of γ- TiAl alloys. Based on the studies broadly available in the literature, it is clear that the ML approach is an economical and reliable strategy to predict the properties of materials and predict the elastic constant. Consequently, utilizing a newly produced dataset, three ML models are employed in this study to estimate the elastic constants of RCCAs. Then, the predicted elastic constants of RCCAs are compared with the available reports from the first principles and experimental methods. This research demonstrates the application of the ML as a high-throughput screening tool for designing RCCAs.

## 2. Data Generation and ML Models

### 2.1. Calculation of CALPHAD and Data Generation via DFT

**Figure 1:**
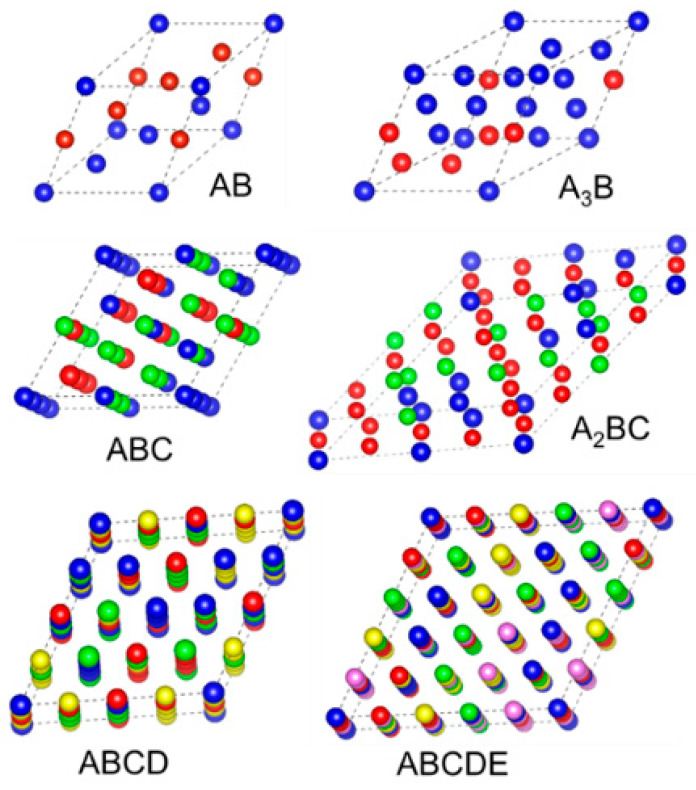
Different SQS models used to calculate the elastic constants of RCCAs.

The database contains binary, ternary, quaternary, and quinary RCCAs having BCC structures with refractory elements of Cr, Hf, Ta, Ti, Mo, Nb, W, Re, Zr, and V. The thermo-calc-2019 package with ThermoCalc’s high-entropy alloy database [35] is employed to identify the RCCAs with stable BCC phases. The predicted phases by the Thermo-Calc package are under the experimental reports [36,37,38,39]. The database for RCCAs with stable BCC phases was created by computing the elastic constants of alloys using special quasi-random structure (SQS) model [40] and the MedeA 2.22 software, Materials Design, Inc., San Diego, CA, USA [41]. The possible alloy supercell structures include AB, A_3_B, ABC, A_2_BC, ABCD, and ABCDE, where each A, B, C, D, and E represents one of the above refractory elements. The diagrams of different SQS structures of RCCAs used in the dataset are shown in Figure 1. The number of atoms of AB, A_3_B, ABC, A_2_BC, ABCD, and ABCDE supercell structures is 8, 16, 36, 32, 64, and 125, respectively. To calculate the elastic constants, i.e., C_11_, C_12,_ and C_44_ of RCCAs, the Vienna Ab initio simulation package (VASP) [42] was used, and the first-principles DFT [43,44] simulations were carried out. The generalized gradient approximation (GGA) was utilized as an exchange-correlation function to simulate many-body electronic interactions, as implemented in the Perdew–Burke–Ernzerhof (PBE) [45]. The electron-ion interactions were detailed using the projector augmented wave (PAW) model [46]. In all calculations, a 500 eV energy cutoff was employed for the plane-wave basis. The requirements for energy and force convergences in relaxation processes are set to be 10^−5^ eV/atom and 10^−5^ eV/Å, correspondingly. A smearing parameter of 0.2 eV was used in the Methfessel–Paxton method [47]. The stability of RCCAs was checked by the formation energy, $E_{f}=E\left(RCCA\right)-\sum_{i}n_{i}\mu_{i}$, where E(RCCA) is the energy of RCCA, $n_{i}$ is the number of atoms of species *i*, and $\mu_{i}$ is the chemical potential of species *i*. The elastic constants of 370 RCCAs are computed from the DFT calculations. By utilizing strain matrix e, altering the Bravais lattice vectors *R* = (a, b, c) to *R*′ = (a′, b′, c′), a deformation of the unit cell is generated, and the elastic constants are obtained,

(1) $$R\prime =R^{}\left(\begin{array}{ccc} 1+e_{xx} & \frac{1}{2}e_{xy} & \frac{1}{2}e_{xz}\\ \frac{1}{2}e_{yx} & 1+e_{yy} & \frac{1}{2}e_{yz}\\ \frac{1}{2}e_{zx} & \frac{1}{2}e_{zy} & 1+e_{zz} \end{array}\right)$$

Due to the deformation, total crystal energy will change. The Le Page and Saxe [48] symmetry-general approach is used for calculating elastic constants from total energy calculations,

(2) $$U=\frac{E_{total}-E_{0}}{V_{0}}=\frac{1}{2}\sum_{i=1}^{6}\sum_{j=1}^{6}C_{ij}e_{i}e_{j}$$

where $E_{0},$ $V_{0},\;\mathrm{and}\;C_{ij}$ represent the total energy of unstrained lattice, volume of the undistorted cell, and elements of the elastic constants matrix, respectively. The unit cell’s symmetry can lessen the number of independent elastic constants, and it is only 3 for the cubic system, i.e., C_11_, C_12_, and C_44_.

### 2.2. Descriptor Selection

Selecting the meaningful physical descriptors is a critical component of an ML algorithm, and the optimal choice usually depends on the target variable under investigation. It is essential to select the descriptors whose value can be predicted with any DFT computations to save computational costs. Initially, ten numeric descriptors of RCCAs and one categorical data (name of alloys) are chosen for ML models, and numeric descriptors are calculated using the Python program. The mechanical and thermal characteristics of the alloy’s elemental constituents are used to calculate these descriptors. The RCCAs descriptors are entropy of mixing (Δ*S_mix_*) [49], enthalpy of mixing (Δ*H_mix_*) [50], the temperature of melting, average atomic radius ($\bar{r}$), average electronegativity ($\bar{\chi }$) and a dimensionless parameter omega (Ω). The Ω is defined as Δ*S_mix_* divided by the Δ*H_mix_* and multiplied by alloys’ average melting temperature (MT) [51]. Material properties cannot be characterized by averaging the constituent properties. Thus, it is imperative to consider the differences in properties [52]. As a result, the characteristic of these differences, i.e., atomic size difference (δ), electronegativity difference (Δ*χ*), and valence electron concentration (*VEC*) of RCCAs [53] are also taken into consideration. The number of descriptors used in these calculations is successfully applied in predicting the stability and properties of alloys in past studies [19,20,21,22,23,24,25,26]. For example, the Islam group [20] used 118 instances of data set with five descriptors, i.e., *VEC*, Δ*S_mix_*, Δ*H_mix_*, δ, and *χ*, to determine the phase in multi-principle element alloys. Khakurel et al. [27] used some ML models to estimate Young’s modulus of CCAs. They found that *VEC*, geometrical parameter, δ, and melting temperature play essential roles in determining the modulus of CCAs. The geometrical parameter (λ), which is defined by Δ*S_mix_* divided by the square of δ, has also been added as a descriptor in this work. The above-selected ten numeric descriptors are calculated using the following Equations (3)–(12), shown in Table 1. A total of 370 alloys used in the ML models are included in the form of categorical data.

In equations of Table 1, $a_{i}{\;\mathrm{and}\;a}_{j}$ are the atomic percentages of the i^th^ and j^th^ element. $r_{i\;}$, $\chi_{i}$ and ${\left(\mathrm{VEC}\right)}_{i}$ are the radius, Pauling electronegativity, and valence electron concentration of the i^th^ element. $\bar{r}$ is averaged atomic radius, $\bar{\chi }$ is average Pauling electronegativity, $T_{m}\;$ is the melting temperature of the element, and R is the ideal gas constant. The required data of refractory elements for calculating the descriptors are shown in Table 2 and Table 3.

### 2.3. Machine Learning Models

**Figure 2:**
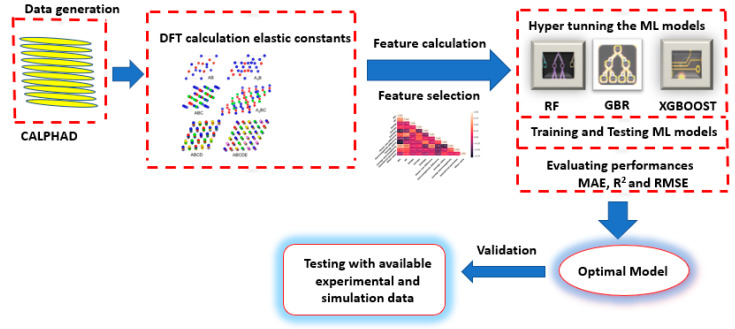
Graphic representation of the workflow process of the current study.

There are 370 RCCAs data points from the DFT calculation. A total of 90% of data were utilized for training, and 10% were used to test and validate the results. The three ML models used in this work are random forest (RF) regressor, gradient boosting regressor (GBR), and XGBoost (XGB) through the Scikit-learn library [55], which is also used for data preparation and model assessment. The RF model is an ensemble approach consisting of many decision trees to improve prediction accuracy and reduce overfitting by averaging the trees. The samples of the training dataset may reappear in the single tree due to sampling replacement, also known as bootstrapping. GBR and XGB are multi-purpose machine learning methods that can handle both classification and regression problems. The GBR is a type of ensemble model having an iterative collection of tree models, and it is capable of learning from the previous model’s mistakes. The XGBoost [56] is a powerful machine learning method that makes a quick decision by implementing boosted decision trees efficiently and effectively. After finalizing the descriptors, the 3 ML models were trained using 370 datasets of elastic constants with all calculated descriptors. The GridSearchCV function in the sklearn library was used to improve the machine learning model with a cross-validation score of 5. The adjustment of hyperparameters was essential since it impacts the overall performance of machine learning algorithms as it cross-validates the training and testing datasets. Following the searching algorithm of GridSearchCV, hyper-tuned parameters for all models were found. It was used in the model to make ML models to predict elastic constants. The root-mean-squared error (RMSE), the average coefficient of determination (R^2^), and mean absolute error (MAE) are calculated for each ML model to evaluate its performance. The most satisfactory model with high performance during the training and testing phases is further used to predict the elastic constants of four experimental and simulation data. These data are entirely new to the model to predict elastic constants. The workflow of this work, as explained above, is illustrated in Figure 2.

## 3. Results and Discussion

### 3.1. Feature Selection Criteria

**Figure 3:**
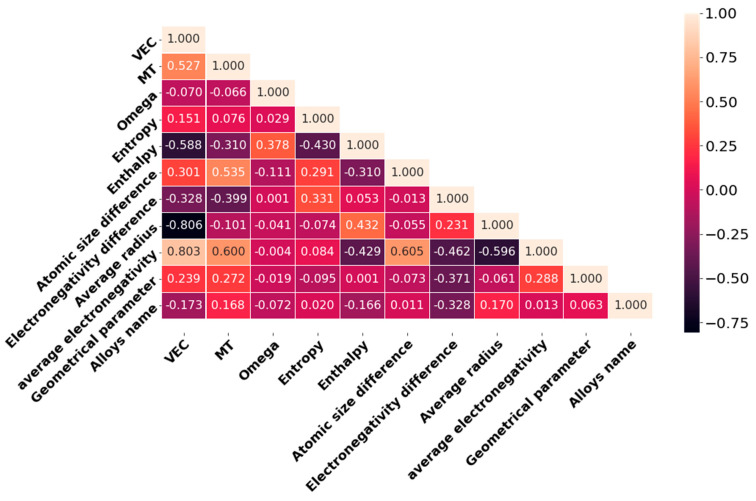
Lower triangular heatmap diagram of 11 descriptors of the initial datasets.

Before applying the ML models, the Pearson correlation coefficients (p) between each pair of descriptors are calculated, and the results are shown using the triangular heatmap in Figure 3. The numeric values of p equal to 0, 1, and −1 denote no correlation, high positive correlation, and high negative correlation, respectively. The highly correlated features are likely to overfit the ML model, which is undesirable. The dark purple and light-pink colors represent highly positive and negative correlations, respectively. Two of the descriptors, i.e., average electronegativity and average radius, are strongly correlated with *VEC*, and it has been removed from the descriptor list. Since there is no substantial dependency between any two of the remaining descriptors, all metrics should be incorporated into the ML model. Therefore, the number of descriptors reduce from 11 to 9.

### 3.2. ML Model Performance

The elastic constant for RCCAs was predicted from the dataset by employing three ML methods: RF, GBR, and XGBoost. The dataset is randomly divided into a training and a testing set with a 9 to 1 ratio. For each approach, hyper tunning is done with a cross-validation score of 5. The different tuned hyperparameter of three ML models obtained from the Grid search method is shown in Table 4. MSA, RMSE, and $R^{2}$ are calculated for measuring the performances of the ML models using the equations below.

(13) $$\mathrm{MAE}=\frac{1}{n}\sum_{i=1}^{n}\left|y_{i}-{\hat{y}}_{i}\right|$$

(14) $$\mathrm{RMSE}=\sqrt{\frac{\sum_{i=1}^{n}{\left(y_{i}-\hat{y}\right)}^{2}}{n}}$$

(15) $$R^{2}=1-\frac{\sum_{i}{\left(y_{i}-\hat{y}\right)}^{2}}{\sum_{i}{\left(y_{i}-\bar{y}\right)}^{2}}$$

where $y_{i}$, ${\hat{y}}_{i}$,$\;\bar{y}$ and *n* represent the DFT elastic constants, ML-predicted elastic constants, mean of all elastic constants, and sample size, respectively.

**Figure 4:**
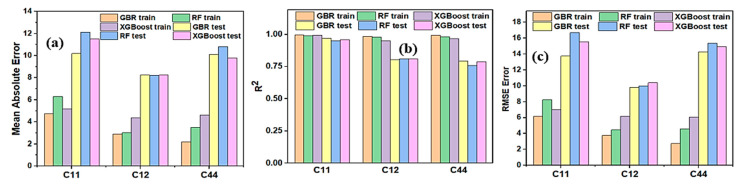
(**a**–**c**) Represents the MAE, R^2^, and RMSE of different ML models used, respectively.

The performance metrics MAE, R^2^, and RMSE of all three models are shown in Figure 4. To evaluate which model performs very well while predicting the elastic constant, MSA, $R^{2}$, and RMSE needs to be analyzed. ML model with a higher value of R^2^ close to 1.0 with low MAE and RMSE will result in better performance. It is hard to distinguish which model performs well on the dataset if only R^2^ is analyzed. Analyzing results from Figure 4a–c, GBR has the least MAE and RMSE with higher R^2^ value than the other two ML models indicating its higher prediction accuracy of elastic constant. The R^2^ value for all models ranges from 0.76–0.97 for the training and testing datasets. Similar value of R^2^ was found by Trostianchyn et al. [57] while predicting the magnetic remanence of Sm-Co magnets using machine learning methods. They found the R^2^ value of their ensemble ML model greater than 0.7. In this work, considering the different performance metrics of the GBR among other models, it is considered to be the optimal model.

**Figure 5:**
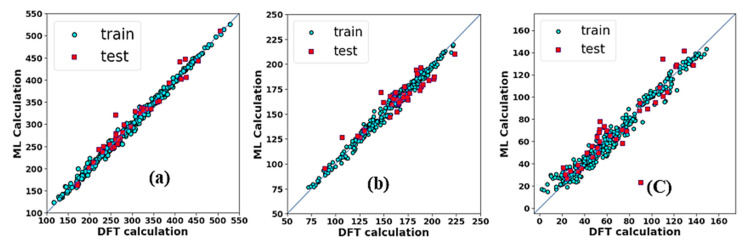
ML-predicted elastic constants of (**a**) C11, (**b**) C12, and (**c**) C44, as a function of DFT-predicted elastic constant during training and testing datasets.

The GBR model optimizes the bias while training the dataset and repeatedly trains the trees in the entire process to avoid the early paralysis of trees during the training data which results in better performance in prediction. The performance metrics of all 3 ML models are listed in Table 5. The MAE of training data of the GBR model is below 5, which means it has predicted the elastic constants of RHEAs with an average error of less than 5 GPa. Similarly, the MAE of the test data of the GBR model is less than 11 for the test dataset, which means there is not much difference in the prediction of the training and testing datasets. This manifests the testing data is not highly overfitted. The overall performances of all three models were good, which confirms that the selection of 9 descriptors fitted well for the ML models. The elastic constants predicted by the optimal GBR models versus DFT results on the training and testing datasets are illustrated in Figure 5. Most training data points (blue dots), and testing data points (dark pink diamonds) are clustered around the light blue solid diagonal line, indicating that predicted elastic constants of RCCAs predicted by the GBR ML model are consistent with the DFT calculations. Some testing data points are slightly far away from the diagonal line. It is due to the absence of sufficient data points around the relevant area. In the future, the addition of more data points will further improve the model, and data points will likely distribute evenly around the diagonal line.

### 3.3. Additional Testing on The Final Model

**Figure 6:**
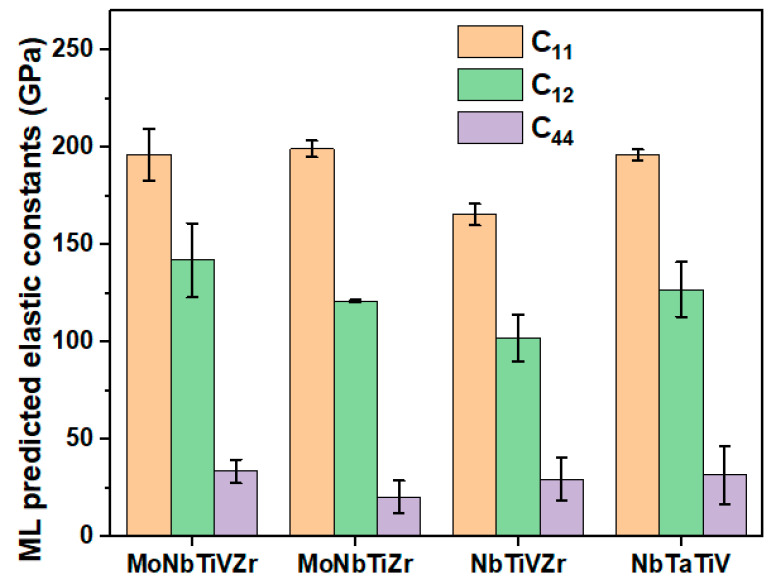
Elastic constants (C_11_, C_12_, and C_44_ are in unit GPa) for MoNbTiVZr, MoNbTiZr, NbTiVZr, and NbTaTiV refractory alloys predicted by the GBR model. The error bar shows how far the elastic constant value has deviated from its original value.

Furthermore, four experimental and simulation data of RCCAs available in the previous publications [10,17] were passed in the optimal model, i.e., GBR, to predict the elastic constants for additional testing. All the elastic constants data of four alloys is provided in Table 6 and the results are shown in Figure 6, a histogram with an error bar. The error bar denotes the deviation from the original prediction. The elastic constants predicted by the GBR ML models for TiZrVNbMo, TiZrNbMo, and TiZrVNb RHEAs are compared with the simulation conducted by Tian’s group. The elastic constants predicted by the ML models are in good agreement with the SQS model calculation since the average error bar length is smaller. However, there is a slight difference between the SQS model and the ML model, which may have been caused by the number of atoms used in the model construction and the applied DFT method. The SQS structures for RHEAs of MoNbTiZr, MoNbTiVZr, and NbTiVZr consist of 128, 250, and 128 atoms, respectively. In comparison, depending on the types of RCCAs, the DFT calculation utilized in this work to build the database comprises supercells of 8, 16, 32, 36, 64, and 125 atoms. Due to inconsistency in the size of the atomic model and applied potential, there is a difference between chemical interactions of atoms in the DFT method, which results in a slight difference in elastic constants [58]. The elastic constant C_11_ predicted by the ML model for NbTaTiV matches the experimental value reported in Lee’s study very well [17]. The slight difference between the ML and the experimental values of elastic constants may result from the temperature difference. The DFT models were performed at 0K, whereas the experiment was conducted at 300 K. Similar results were found while predicting the elastic constants of HEA Al0.3CoCrFeNi by Kim et al. [28]. The predicted elastic constants of HEA Al0.3CoCrFeNi deviate from the measured values from the in situ neutron-diffraction. As mentioned before, measuring elastic constants by experiment is time consuming and has its limitation. It is expected more high throughput experimental methods will be designed to confirm the findings of the elastic constants for RHEAs MoNbTiVZr, MoNbTiZr, and NbTiVZr.

### 3.4. Descriptor Importance and Visualization

**Figure 7:**
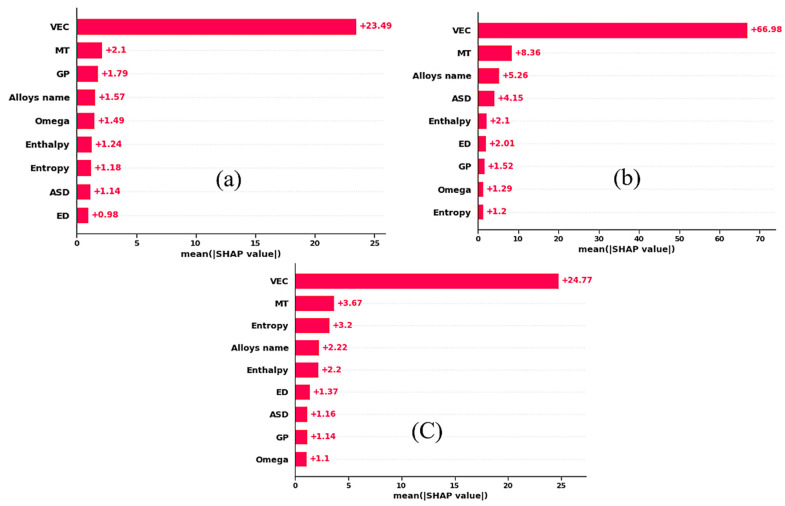
(**a**–**c**) Shows the SHAP value for C_11_, C_12_, and C_44,_ respectively.

The Shapley Additive exPlanations (SHAP) method explains how each descriptor influences the ML by calculating the Shapley values [59]. All the dataset instances are used in the SHAP bar graph by taking the mean absolute value of every descriptor. The relevance ranking of 9 descriptors and their role in contributing to the elastic constants are displayed in Figure 7. *VEC* and MT hold the top two positions in predicting the elastic constants. From the previous findings, it is interesting to note the role of the *VEC* descriptor, which has the top position in descriptor importance in determining the crystal structure [20,21,22,23,24,25], Young’s modulus [27,60,61], and hardness [62] of HEAs. The previous study has shown that *VEC* is the essential feature in determining Young’s modulus of complex concentrated alloys in their ML models [27]. Moreover, Roy et al. [63] also found that MT is an essential parameter in predicting Young’s modulus of HEAs using the gradient boosting method.

**Figure 8:**
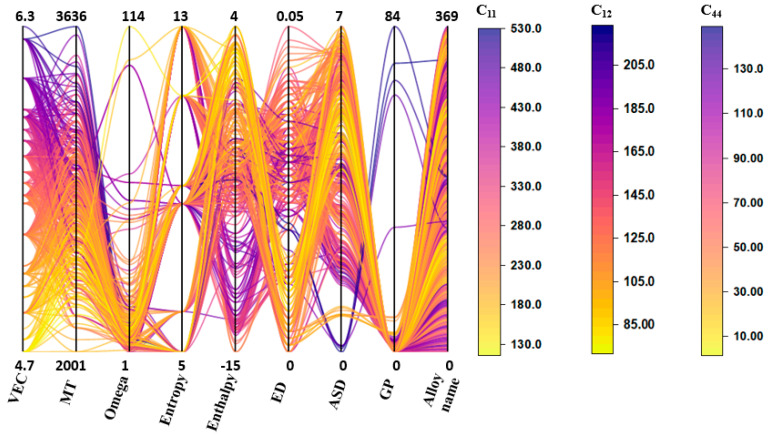
Parallel coordinate plots of elastic constants (C_11_, C_12_, and C_44_ are in GPa) from the datasets.

**Figure 9:**
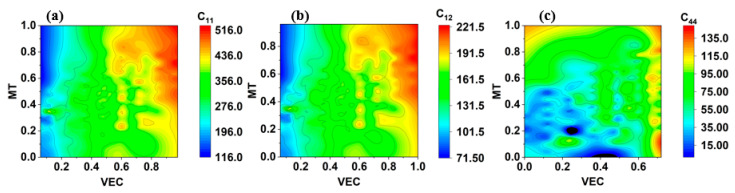
(**a**–**c**) Represents the contour plot of elastic constants C_11,_ C_12_, and C_44_, respectively.

Using the SHAP value, the relevance ranking of various descriptors provides a numerical correlation between the input descriptors and target descriptors. Further details between the descriptors and elastic constant can be explored using the data visualization techniques and the concept of material. The parallel coordinate plot (PCP) of the dataset is plotted to display the correlations of the descriptor with the elastic constants, and it is shown in Figure 8. The highest value of each descriptor is shown on the top and bottom of the vertical axes, respectively. The values of alloy elastic constants are demonstrated by the three-colored bars on the right-hand side of Figure 8. The purple color represents the highest elastic constants, and yellow represents the lower elastic constant. The PCP indicates that higher *VEC*, MT, and GP values with lower omega values are favorable for developing a higher elastic constant in refractory alloys. The *VEC* value positively influences the elastic constant of RHEAs; the greater the *VEC*, the higher the elastic constant, and vice versa. This observation is quite similar to Guo et al. findings [53], which show when *VEC* is less than 6.87, the solid solution phase of the BCC is more stable due to solid strengthening caused by lattice distortion. Similar phenomena is observed from the PCP, when *VEC* lies between 5 to 6.3, the elastic constant becomes higher and it could be due to the lattice distortion. The lattice distortion is also formed when different size elements are mixed, and this mixing will force the atoms to move from their original position and result in lattice distortion. Previous work has also described the significance and importance of *VEC* in physical characteristics and phase stability in alloys [53,54,64,65]. Similar results were found by Huang et al. [66] about the *VEC*, which holds the top position in features importance while predicting the hardness of RHEAs using the RF model. They also highlight that *VEC* is important in charge transfer, which impacts RHEA strength because of lattice distortion. So far, the lattice distortion study is still in progress, and it is done by simulation only. Experimental measurement of lattice distortion in alloys is needed to get further knowledge about the contribution of each element to the elastic properties of RHEAs. The second top descriptor is melting temperature. According to the PCA plot, RHEAs with higher melting temperatures favor the elastic constant of materials. The RHEAs consisting of elements Re, W, Ta, and Mo which have higher *VEC* and MT, have increased the alloy’s elastic constant. Additional visualization using the contour plot is plotted to see the relationship between top important features *VEC* and MT with elastic constants. Figure 9a–c show the contour plot of the elastic constants of RHEAs as a function of *VEC* and MT. The original value of *VEC* and MT is normalized to get the smooth graph. The right-hand side color bar denotes the values of elastic constant in GPa. According to the contour plot, elastic constants of RHEAs increased with higher MT and *VEC* values. If MT is higher in RHEAs, with low *VEC*, then the alloy will have low elastic constants. A similar color pattern graph is obtained for all elastic constants as a function of *VEC* and MT. As a result, it is necessary to consider MT and *VEC* to get high elastic constants. The change in elastic constants with change in materials stability will help to improve the mechanical properties.

### 3.5. Comparison of Current Work with Previous Work

In terms of elastic constant, Vasquez et al. [67] reported the analytical model to predict the elastic constants of RHEAs consisting of five elements Nb, Mo, Ta, W, and V alloys. In comparison, ten elements were included in the dataset in this study to give a boarder range of compositional space for alloys. Furthermore, hyper tunning of all 3 ML models is also performed, and metrics such as MSE and RMSE are calculated to find the optimal ML model capable of predicting elastic constant with higher accuracy. The dataset and model could be more beneficial to optimizing the various alloys and help in alloy design.

As the availability of elastic constants for RCCAs is very limited, this study demonstrates the methodology of estimating the elastic constants for RCCAs based on the ML models using a database constructed from DFT calculations. A total of 370 instances of data were created with the help of DFT computational simulations. The used database is relatively small, but as previous studies show, ML can successfully be applied to a small dataset. As an example, Islam et al. [20] also used 118 instances and five features of the multi-principal element alloys dataset, which were used to forecast the alloy’s structure. Wen et al. [32] applied ML methods to the alloy system of Al-Co-Cr-Cu-Fe-Ni to find the maximum hardness with just 155 samples. Another group [68] identified the phase formation in HEAs with a small dataset by selecting low dimension descriptors. This study aimed to predict the elastic constants of RCCAs without performing any expensive DFT simulations and experimental methods, which is supposed to save time, effort, and money in designing RCCAs. It is indispensable to state that elastic constants are essential properties affecting materials’ stability, strengths, and anisotropy. There is still plenty of room to optimize the compositional space of alloys. It is necessary to determine the shear strains using an atomic displacement field to evaluate the lattice distortion. The elastic constant is also one parameter for obtaining the overall influence of such local strain changes [69]. Therefore, faster methods that are effective in exploring the compositional space of alloys for creating high-temperature and high-pressure performance materials are highly demanded. The current ML methods can acquire information from the descriptor of datasets and link the relationship between the HEAs composition and target properties, i.e., elastic constants. One achievement of this study is that if a user comes up with a new composition of an alloy, the provided Python program can predict all of the nine descriptors for the considered alloys. If the obtained information is passed into the trained ML models, the elastic constant of the given alloy will be predicted. These models may be further applied to study the compositional space of novel RCCAs, and they can be used to develop future RCCAs with desirable mechanical properties. These ML models do not consider complex phenomena such as lattice defects, dislocations, grain boundaries, slip planes, and plastic deformation in RCCAs. It is expected that more powerful models will be developed that can consider the complex phenomenon of RCCAs to make more accurate predictions of mechanical properties.

## 4. Conclusions

In this study, three popular ML methods, namely random forest regressor, gradient boosting regressor, and decision tree regression models, were used for predicting the elastic properties of RCCAs. The ML model consists of eight different descriptors of RCCAs. GBR performs very well as compared with the other two ML models. While predicting elastic constants C_11_, C_12_, and C_44_, the R^2^ value of testing data of the GBR model were 0.97, 0.803, and 0.787, respectively. The GBR model has a high R^2^ value and low MAE and RMSE error than RF and XGB ML models and is promising for predicting the elastic constant of RHEAs. The elastic constants of alloys MoNbTiVZr, MoNbTiZr, NbTiVZr, and NbTaTiV predicted by the GBR model are consistent with the current simulation and experimental findings, which validate its prediction accuracy and applicability. The feature importance from SHAP and PCP plot shows that the *VEC* and MT are the most influential features affecting the elastic constants of RHEAs. The dataset created in this study using CALPHAD and DFT modeling could help scientists to construct RHEAs that can endure extreme temperatures and pressures. Compared to the computationally expensive DFT calculation and experimentally tedious trial-and-error methods for calculating the elastic constants, ML methods can be applied to predict the elastic constant of any novel refractory high-entropy alloys with very low costs, and hence helps on designing RHEAs to meet the industry demands. The GBR model is not capable of predicting the elastic constants of RHEAs at different temperatures and therefore future work should be focused on creating an ML model that can predict the elastic constants at varying temperatures.

## Figures and Tables

**Table 1:** List of the mathematical formulation of descriptors used for different ML models.

Descriptors	Abbreviation	Descriptors Calculation Formula
Entropy of mixing	Entropy	(3) ${\Delta S}_{\mathrm{mix}}=-R\;\sum_{i=1}^{m}a_{i}{\mathrm{lna}}_{i}$
Enthalpy of mixing	Enthalpy	(4) ${\;\Delta H}_{\mathrm{mix}=}\sum_{i=1,i\neq j\;}^{m}4{\Delta H}_{\mathrm{ij}}^{\mathrm{mix}}a_{i}a_{j}$
Melting temperature	MT	(5) ${\;T}_{m}=\sum_{i=1}^{m}a_{i}{\left(T_{m}\right)}_{i\;}{}_{\;}$
Average atomic radius	AAR	(6) $\bar{r}=\sum_{i=1}^{n}a_{i}r_{i}$
Average electronegativity	AE	(7) $\bar{\chi }=\sum_{i}^{n}a_{i}\left(\chi_{i}\right)$
Unitless parameter Omega	Omega	(8) $\Omega \;=\frac{T_{m}{\Delta S}_{\mathrm{mix}}\;}{\left|{\Delta H}_{\mathrm{mix}}\right|}$
Atomic size difference	ASD	(9) $\delta =100\times \sqrt{\sum_{i=1}^{m}a_{i}{\left(1-\frac{r_{i}}{\bar{r}}\right)}^{2}}\bar{r}$
Valence electron concentration	*VEC*	(10) $\mathrm{VEC}=\sum_{i=1}^{m}a_{i}{\left(\mathrm{VEC}\right)}_{i}$
Electronegativity difference	ED	(11) $\Delta \chi =\sqrt{\sum_{i}^{n}a_{i}\left(\chi_{i}-\bar{\chi }\right)}$
Geometrical parameter	GP	(12) $\lambda =\frac{{\Delta S}_{\mathrm{mix}}}{\delta^{2}}$

**Table 2:** The atomic radius, *VEC*, Pauling electronegativity, and Melting Temperature for refractory elements [54].

Element	Radius (Å)	*VEC*	Pauling Electronegativity	Melting Temperature (Kelvin)
Cr	1.249	6	1.66	2180
Hf	1.578	4	1.3	2506
Mo	1.363	6	2.16	2896
Nb	1.429	5	1.6	2750
Re	1.375	7	1.9	3459
Ta	1.43	5	1.5	3290
Ti	1.462	4	1.54	1941
V	1.316	5	1.63	2183
W	1.367	6	2.36	3695
Zr	1.603	4	1.33	2128

**Table 3:** The binary alloys’ mixing enthalpies ∆Hmix (kJ/mol) [47].

Element	Cr	Hf	Mo	Nb	Re	Ta	Ti	V	W	Zr
**Cr**	0	−9	0	−7	−4	−7	−7	−2	1	−12
**Hf**	−9	0	−4	4	−2	3	0	−2	−6	0
**Mo**	0	−4	0	−6	−7	−5	−4	0	0	−6
**Nb**	−7	4	−6	0	−26	0	2	−1	−8	4
**Re**	−4	−30	−7	−26	0	−24	−25	−13	−4	−35
**Ta**	−7	3	−5	0	−24	0	1	−1	−7	3
**Ti**	−7	0	−4	2	−25	1	0	−2	−6	0
**V**	−2	−2	0	−1	−13	−1	−2	0	−1	−4
**W**	1	−6	0	−8	−4	−7	−6	−1	0	−9
**Zr**	12	0	−6	4	−35	3	0	−4	−9	0

**Table 4:** Tuned hyperparameter obtained from Grid Search method.

Elastic Constant	ML Models	Hyper-Tuned Hyperparameters
	GBR	Learning rate: 0.01, max depth: 8, estimators: 900, subsample: 0.2
C_11_	RF	Max depth: 10, estimators: 1000
	XGB	Learning rate: 0.2, estimators: 100
	GBR	Learning rate: 0.04, max depth: 4, estimators: 200, subsample: 0.7
C_12_	RF	Max depth: 10, estimators: 500
	XGBoost	Learning rate: 0.1, estimators: 100
	GBR	Learning rate: 0.01, max depth: 6, estimators: 900, subsample: 0.3
C_44_	RF	Max depth: 10, estimators: 500
	XGBoost	Learning rate: 0.1, estimators: 100

**Table 5:** Different metrics of the dataset using different ML models.

Elastic Constant	ML Models	R^2^ Train	R^2^ Test	MAE Train	MAE Test	RMSE Train	RMSE Test
	GBR	0.995	0.97	4.74	10.24	6.17	13.7
C_11_	RF	0.995	0.95	6.31	12.17	8.25	16.69
	XGBoost	0.994	0.964	5.2	11.52	6.99	15.5
	GBR	0.985	0.803	2.19	8.25	3.77	9.88
C_12_	RF	0.979	0.815	3.032	8.2	4.46	10.10
	XGBoost	0.959	0.812	4.392	8.23	6.16	10.39
	GBR	0.994	0.787	2.20	10.38	2.67	14.45
C_44_	RF	0.981	0.761	3.58	10.86	4.62	15.4
	XGBoost	0.967	0.787	4.622	9.84	6.06	14.95

**Table 6:** Elastic constants (C_11_, C_12_, and C_44_ are in GPa) for MoNbTiVZr, MoNbTiZr, NbTiVZr, and NbTaTiV refractory alloys experimental (exp.), and SQS.

Elastic Constants of RHEAs	MoNbTiVZr (SQS)	MoNbTiZr (SQS)	NbTiVZr (SQS)	NbTaTiV (exp)
C_11_	209.3	203	159	196
C_12_	123	121	114	121
C_44_	39.12	29.6	18.5	46

SQS [10] exp. [17].

## Data Availability

The dataset utilized to produce the results in this work is accessible at https://github.com/uttambhandari91/Elastic-constant-DFT-data.
